# Screening and identification of novel protein markers of early-stage lung cancer and construction and application of screening models

**DOI:** 10.3389/fonc.2025.1567673

**Published:** 2025-05-27

**Authors:** Huijie Yuan, Shuyin Duan, Clement Yaw Effah, Sitian He, Yaru Chai, Xia Liu, Lihua Ding, Yongjun Wu

**Affiliations:** ^1^ College of Public Health, Zhengzhou University, Zhengzhou, China; ^2^ School of Public Health, Shandong First Medical University & Shandong Academy of Medical Sciences, Jinan, China

**Keywords:** lung cancer, screening, protein markers, machine learning, high-risk individuals

## Abstract

**Objective:**

Molecular biomarkers have the potential to improve the current state of early screening of lung cancer. This investigation aimed to identify novel protein markers for early-stage lung cancer and combine them with traditional tumor markers to develop machine learning models for lung cancer screening.

**Materials and methods:**

The protein alters of peripheral blood (5 patients with early-stage lung adenocarcinoma, 5 patients with early-stage lung squamous cell carcinoma, and 8 healthy controls) were detected by label-free quantitative proteomics. The novel candidate protein markers were preferentially selected by multi-omics technology. Then, the malignant transformation of BEAS-2B cells and lung carcinogenesis in C57BL/6 mice were induced by coal tar pitch extracts (CTPE) so that the expressions of these markers at different stages of lung carcinogenesis could be dynamically tracked and validated. These markers in human plasma were detected and further confirmed by ELISA. Machine learning models were established to screen high-risk individuals of lung cancer.

**Results:**

The C-type lectin domain family 3 member B (CLEC3B), membrane primary amine oxidase (AOC3), hemoglobin subunit beta (HBB), catalase (CAT), and selenoprotein P (SEPP1) were screened as candidate protein markers for early-stage lung cancer. The expressions of CLEC3B, AOC3, CAT, and SEPP1 were statistically significant in various passages of cells cultured with exposure to CTPE compared to the saline group (*P*<0.05). In addition, the expressions of these 5 proteins were statistically significant in lung tissues, plasma, and alveolar lavage fluid of mice exposed to CTPE for 3, 6, 9 and 12 months compared to normal controls (*P*<0.05). There were notable variations in AOC3, CAT, CLEC3B, SEPP1, HBB, CEA, CYFRA21-1, and NSE among the healthy control group, lung cancer group and coke oven workers (*P*<0.05). The decision tree C5.0 (AUC=0.868) and artificial neural network (AUC=0.844) which combined these 8 markers showed better performance.

**Conclusion:**

The differential changes of AOC3, CAT, CLEC3B, SEPP1, and HBB protein were proven as early molecular events in lung tumorigenesis. The screening models of lung cancer based on the novel protein markers and traditional tumor markers might be applied for the screening of high-risk individuals.

## Graphical Abstract

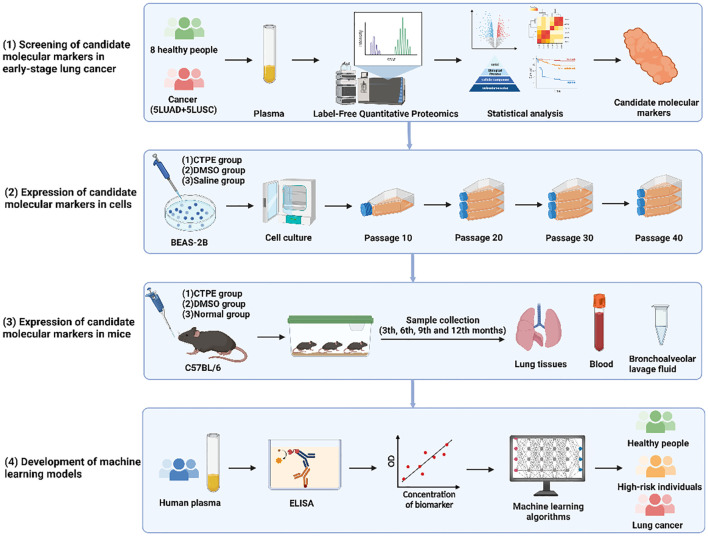

## 1 Introduction

The global burden of cancer is rising due to population aging and increasing pollution (1, 2). Lung cancer ranks second in incidence and remains the leading cause of cancer-related deaths worldwide (3). Its development involves multiple factors, genes, and stages (4). Occupational exposure is strongly associated with lung cancer (5). Coal tar pitch (CTP) is a known human carcinogen (6), and long-term exposure to coal tar pitch extracts (CTPE) can induce chromosomal abnormalities and oxidative stress, contributing to tumorigenesis (7, 8). Due to the lack of efficient method for early screening, most lung cancer patients are diagnosed at advanced stages with poor survival outcomes. Early identification and standardized treatment can significantly improve prognosis (9). Therefore, greater attention should be focused on the development of screening strategies for the early detection of lung cancer.

Low-dose computed tomography (LDCT) is widely used for the early screening of lung cancer (10), but challenges such as high false-positive rates, radiation exposure, and overdiagnosis remain unresolved (11). Thus, early screening for lung cancer remains a huge challenge for clinicians, and identifying molecular markers with high specificity and sensitivity could help address this issue. As the carrier of human life, proteins have emerged as important players affecting all life activities. In addition, non-coding RNAs, such as miRNA, lncRNA and circRNA mediate intercellular communication by regulating protein expression (12). Accordingly, circulating proteins in blood remain the most promising biomarkers for early-stage lung cancer. In clinical application, protein molecules such as cytokeratin 19 fragment (CYFRA21-1), carcino-embryonic antigen (CEA) and neuron-specific enolase (NSE) are commonly used for the diagnosis of lung cancer. However, the sensitivity and specificity are low, limiting their diagnostic value for lung cancer (13). Advances in mass spectrometry have facilitated the discovery of novel protein markers. Nevertheless, studies on the screening of markers by proteomics have included limited samples of early-stage lung cancer, and these markers have rarely undergone multiple validations *in vivo*, *in vitro*, and across large populations (14, 15). Hence, it is necessary to identify and validate novel circulating protein markers at multiple levels to support the screen for early-stage lung cancer.

The rapid growth of biological big data and the establishment of cancer-related databases, such as The Cancer Genome Atlas (TCGA), Kaplan-Meier Plotter and Oncomine, have facilitated the discovery of novel markers for lung cancer (16, 17). While transcriptomics reveals the patterns of gene expression, proteins are the direct executors of cellular function. Integrating proteomics and transcriptomics leverages the complementarity of multi-omics approaches, enabling a more comprehensive analysis of gene expression. Furthermore, machine learning is an emerging field in medicine and is important for a deeper understanding of the classification of disease (18, 19). Machine learning models include support vector machine (SVM), decision tree (DT), and artificial neural network (ANN) (20–22). These approaches support the development of predictive models for lung cancer screening, offering new strategies for early detection.

In this study, we used multi-omics technology to identify novel candidate protein markers in the peripheral blood of patients with early-stage lung cancer. Next, CTPE-induced lung carcinogenesis models in cells and animals were employed to jointly investigate the dynamic changes of candidate markers during early lung carcinogenesis. Then, the sample size of the population was expanded to further validate the expression of candidate protein markers in human plasma samples. Finally, machine learning was used to construct screening models of lung cancer based on the candidate protein markers and traditional tumor markers (CEA, CYFRA21-1, and NSE). These models are expected to provide a new and effective approach to accurately screen for lung cancer and help conserve public health resources. The flowchart of the study was presented in Graphical Abstract.

## 2 Materials and methods

### 2.1 Collection of plasma samples

A total of 185 lung cancer patients and 163 healthy controls were recruited from the First Affiliated Hospital of Zhengzhou University from September 2018 to September 2019. Additionally, 163 coke oven workers were selected in July 2018 from a coking plant of an iron and steel company in Henan, China. In the morning, 5 mL venous blood was drawn from each fasting individual in vacuum tubes with ethylenediaminetetraacetic acid (EDTA) anticoagulant. The samples were then centrifuged at 1500 g for 5 min. Plasma was extracted and preserved at -80°C for subsequent analyses.

The inclusion criteria for lung cancer patients were as follows: (1) a pathologically confirmed diagnosis of primary lung cancer, (2) complete and well-documented clinical information, (3) no prior history of surgical treatment, pharmacological therapy, or radiochemotherapy, (4) no concurrent malignancies in other organs and no previous history of malignancies in other organs, (5) willingness to participate in the study with good compliance. The inclusion criteria for healthy controls were as follows: (1) good overall health status, (2) no history of malignant tumors in the lungs or other organs, (3) willingness to participate in the study with good compliance. Basic demographic and lifestyle data were collected via questionnaire. This study was approved by the Ethics Committee of Zhengzhou University (Grant Number: ZZUIRB2021-106) and informed consents were obtained from all patients.

### 2.2 Detection of plasma proteins and analysis of differential protein expression

Plasma samples were obtained from 5 individuals with lung adenocarcinoma (LUAD) (3 cases in stage I and 2 cases in stage II), 5 individuals with lung squamous cell carcinoma (LUSC) (4 cases in stage I and 1 case in stage II) and 8 healthy volunteers. Label-free quantitative proteomics was used to detect the types and expression of plasma proteins. The mass spectrometric data was analyzed by MaxQuant 1.5.3.17 software. Differentially expressed proteins were identified by screening proteins with a threshold fold change greater than 1.5.

### 2.3 Analysis of transcriptomic data of early-stage lung cancer

The transcriptome sequencing data about lung cancer were downloaded from TCGA database. The sequencing data included 395 patients with early-stage LUAD, 406 patients with early-stage LUSC, and 43 cases of tumor-adjacent tissues.

Statistical models were constructed after log2 transformation of the obtained transcriptome data. Clustering analysis and principal component analysis (PCA) were performed to explore the effect of lung cancer on the expression of genes. The limma package was employed to screen differentially expressed genes (DEGs), and volcano plots were applied to visualize gene expression. The criteria for identifying differentially expressed genes were as follows: *P*<0.05 after Benjamini-Hochberg correction and the fold change (FC) of the level of gene expression ≥ 2. Venn diagrams were used to identify DEGs between LUAD and LUSC.

The Gene Ontology (GO) and Kyoto Encyclopedia of Genes and Genomes (KEGG) were performed for the functional enrichment and the construction of regulatory networks of the DEGs.

### 2.4 Survival analysis

Genes corresponding to the differentially expressed proteins were obtained by aligning with UniProt database. Venn diagrams were applied to determine DEGs between plasma samples and lung cancer lesions. The effect of related genes on the survival time of early-stage lung cancer patients was investigated via the Kaplan-Meier Plotter database. The median gene expression level stratified patients into high or low expression categories. All 240 months follow-up data were included.

### 2.5 Oncomine analysis

By using the Oncomine Platform, the expression of DEGs was assessed in lung cancer tissues and matched to normal adjacent lung tissues. The following standards were applied during the analysis: mRNA as the data type, *P* < 0.05, FC ≥ 2 and top 10% gene rank as threshold.

### 2.6 Preparation of coal tar pitch extracts

Medium temperature CTP was collected from the coking plant in a certain iron and steel company in Henan Province, China. The CTP was grinded into powder and sieved by 200 meshes of 0.074mm in diameter. Then, the fine powder of CTP was put into a beaker and heated at 400°C to collect smoke on a dust sampler with 0.8 µm nitrocellulose filter membranes. After that, the filter membranes were cut into pieces and dissolved into ethyl acetate solution in a stoppered flask by supersonic vibration for 40 min. Finally, the solution was filtered by a sand core funnel and dried in baking oven at 45°C. Dimethyl sulfoxide (DMSO) was used to dissolve CTPE to the final concentration of 10 mg/mL for cell experiments. In addition, CTPE was dissolved in DMSO and corn oil with the final concentration of 20 mg/mL for animal experiments. The composition of CTPE was examined using gas chromatography and mass spectrometry (GC/MS).

### 2.7 Cell culture and CTPE treatment

The BeiNa Culture Collection (Beijing, China) provided the human bronchial epithelial cells (BEAS-2B) and A549 cells, which were cultivated in RPMI 1640 medium containing 10% fetal bovine serum (FBS) under standard culture conditions (37°C and 5% CO_2_). Once the BEAS-2B cells reached 70%–80% confluency, they were washed three times with PBS. Then, 5 mL of culture medium containing 15.04 μg/mL CTPE was added to the flasks for exposure. After 24 hours, the cells were digested with trypsin containing 0.25% EDTA, passaged, and subsequently maintained in normal culture medium. When the cells again reached 70%–80% confluency, the exposure procedure was repeated for another 24 hours. This process was repeated for a total of 5, 10, and 15 cycles. The cells collected immediately after the final exposure were designated as passage 0. Each subsequent passage was numbered sequentially, and the first-passage cells in each treatment group were denoted as CTPE5-1, CTPE10-1, and CTPE15-1, respectively. All groups were continuously cultured up to passage 40. DMSO was used as the vehicle control, and saline solution was used as the negative control. In our preliminary experiments, early signs of cellular transformation were observed in CTPE-exposed cells at approximately passage 10, with progressively pronounced malignant features emerging at later passages (23, 24). Therefore, passages 10, 20, 30, and 40 were specifically selected to represent different stages of cellular transformation, enabling dynamic monitoring of CTPE-induced carcinogenesis.

### 2.8 Plate clone formation assay

In the saline, DMSO or CTPE group, 6-well plate was seeded with 100 cells/well at passage10, 20, 30 or 40, and cultured for 2 weeks under standard culture conditions (37°C and 5% CO_2_). The medium was replaced every 5 days, and the clone greater than 50 cells was counted after 20 min of 95% methanol fixation and 20 min of Giemsa staining.

### 2.9 Xenograft assay

Three to four weeks old NOD-SCID mice were purchased from Beijing Vital River Laboratory Animal Technology Co., Ltd. (Beijing, China). Mice were treated humanely in terms of relieving suffering. First, each nude mouse had its right flanks injected with 200 µL of PBS containing 2 *×* 10^7^ cells at passage 40 in the different mode of CTPE treatments (5 times, 10 times and 15 times). Tumor formation in each mouse was carefully monitored after inoculation. On the 30th day, the tumor was removed and weighed.

### 2.10 Animal maintenance and intervention

One hundred and eighty C57BL/6 mice (half male and female, 6-8 weeks old) were brought from Beijing Vital River Laboratory Animal Technology Co., Ltd. (Beijing, China). Every mouse was kept in a controlled environment, fed a standard chow diet and water. Every attempt was made to reduce the stress and discomfort of animals. All experiments were reviewed and approved by the ethics committee of Zhengzhou University.

The mice were randomly divided into three groups: the CTPE group, the DMSO vehicle control group, and the normal control group, with 60 mice in each group. With reference to our group’s previous research (25), (1) Mice in the CTPE group were intratracheally instilled with 50 μL of CTPE (1 mg per mouse, dissolved in a 1:4 volume ratio of DMSO and corn oil) once a week for four weeks. (2) Mice in the DMSO vehicle control group were administered 50 μL of a mixture of DMSO and corn oil (1:1 volume ratio). (3) Mice in the normal control group were not dosed with any solution.

The mice were then sacrificed in batches at the 3rd, 6th, 9th, and 12th month after being exposed for the first time, and they were weighed before dissection. Following the collection of blood in vacuum tubes containing EDTA anticoagulant, the plasma was extracted by centrifugation at 1500r/min for 5 min and stored at -80°C. The tumors were observed in each mouse, and the diameter of tumor was measured with a vernier caliper. One milliliter of saline was used to lavage the lungs and the lavage fluid were gathered in sterile tubes. The lungs of the mice were removed and weighed. The lung tissues were stained by hematoxylin and eosin (HE) and two experienced pathologists identified the type of pathology.

### 2.11 Western blot

The lung tissue of mice and cells with different interventions were lysed with RIPA lysis buffer with proteinase inhibitors. The total protein of the lung tissue and cells were extracted by centrifuging the total lysate at 12,000 g for 15 min and determined by a BCA kit. The sample loading buffer was combined with the protein and boiled for 5 min. Following their separation using 10% SDS-PAGE, the samples were transferred onto PVDF membranes. Following a 2 h room temperature blocking with 5% non-fat milk, the membranes were coated with specific primary antibodies (AOC3, CAT, CLEC3B, SEPP1, HBB, and GAPDH) overnight followed by 3 rounds of rinsing with TBST. Secondary antibody was applied to the membranes and incubated for 0.5 h at 37°C. Again, the membranes were washed thrice with TBST and reacted with the chemiluminescence buffer. Finally, they were visualized under the Fluor ChemHD2Gel imaging system (ProteinSimple, San Jose, CA, USA). GAPDH level was used as the loading control.

### 2.12 Measurement of 8 tumor markers by ELISA

The levels of AOC3, CAT, CLEC3B, SEPP1, and HBB in the plasma and alveolar lavage fluid of mice were measured using ELISA (Cusabio Biotechnology, Wuhan, China). The levels of CEA, CYFRA21-1, NSE, AOC3, CAT, CLEC3B, SEPP1, and HBB in human plasma were measured by ELISA (Cusabio Biotechnology, Wuhan, China).

### 2.13 Establishment of machine learning models

Four different machine learning models were trained as a binary model to predict cancer or healthy control, according to a 3:1 ratio of training and testing sets. The 5 candidate tumor markers (AOC3, CAT, CLEC3B, SEPP1, HBB) were presented as the input parameters for Fisher-5, C5.0-5, ANN-5, and SVM-5 models. The 3 traditional tumor markers (CEA, CYFRA21-1, NSE) were applied as the input parameters for Fisher-3, C5.0-3, ANN-3, and SVM-3 models. The 8 plasma tumor markers (AOC3, CAT, CLEC3B, SEPP1, HBB, CEA, CYFRA21-1, NSE) were employed as the input parameters for Fisher-8, C5.0-8, ANN-8, and SVM-8 models. The feature extraction of protein molecules was performed using SPSS Clementine 12.0. The configuration parameters of different models were shown below:

1. Configuration parameters of the C5.0 model

Mode name: Auto; Use partitioned data: No; Output type: Decision tree; Group symbolics: No; Use boosting: Yes; Number of trials: 10; Cross-validate: No; Mode: Expert; Pruning severity: 75; Minimum records per child branch: 2; Use global pruning: Yes; Winnow attributes: No; Use misclassification costs: No; Model Evaluation: Calculate variable importance.

2. Configuration parameters of the ANN model

Mode name: Auto; Use partitioned data: Yes; Method: Exhaustive prune; Sample: 50%; Set random seed: No; Stop on: Time(mins)1 min; Optimize: Memory; Continue training existing model: No; Use binary set encoding: Yes; Show feedback graph: Yes; Model selection: Use best network; Mode: Expert; Model Evaluation: Calculate variable importance.

3. Configuration parameters of the SVM model

Mode name: Auto; Use partitioned data: Yes; Mode: Simple.

4. Configuration parameters of the Fisher model

Mode name: Auto; Use partitioned data: No; Method: Enter; Mode: Expert; Prior probabilities: All groups equal; Use covariance matrix: Within-groups; Model Evaluation: Calculate variable importance.

K-fold cross-validation was performed to assess the generalizability of the model to new data.

### 2.14 Statistical analysis

In this study, the sample size was estimated based on the Events Per Variable (EPV) principle, with a minimum of 10 outcome events required per predictor variable (EPV≥10) to ensure the stability and reliability of the model estimation. R software was used for statistical description, comparative analysis and data visualization. SPSS21.0 software was employed for statistical analysis. Continuous variables that followed a normal distribution were expressed as mean ± standard deviation. Comparisons between two groups were conducted using the t-test, while one-way ANOVA was used for comparisons among three groups. For continuous variables that did not follow a normal distribution, data were presented as median (*P*_25_, *P*_75_). The Mann–Whitney U test was applied for comparisons between two groups, and the Kruskal–Wallis H test was used for comparisons among three groups. Categorical variables were compared using the *χ*² test. A *P* value ≤ 0.05 was considered statistically significant.

## 3 Results

### 3.1 The expressions of plasma proteins in lung cancer and normal controls

**Figure 1:**
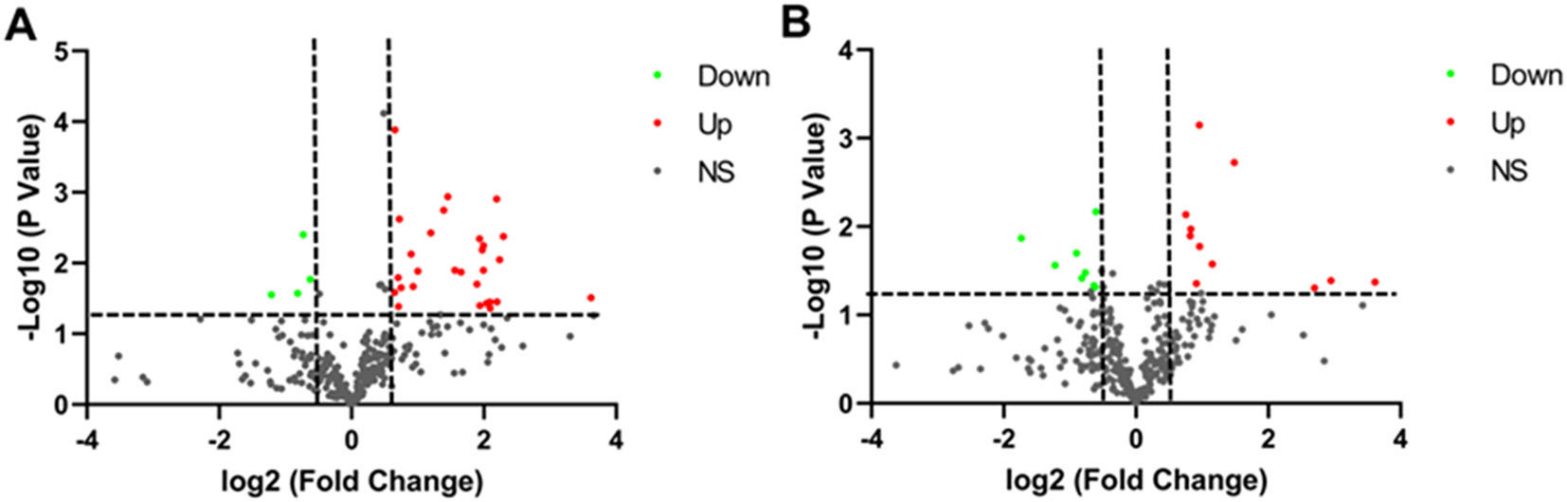
Volcano plot of the differential expression of plasma protein in individuals with lung cancer and healthy controls. **(A)** Volcano plot of differentially expressed proteins in LUAD. **(B)** Volcano plot of differentially expressed proteins in LUSC. Green indicates proteins that are downregulated. Red indicates proteins that are upregulated. Black represents proteins that were not significantly altered.

Plasma samples from 5 LUAD patients, 5 LUSC patients and 8 healthy controls were analyzed using label-free quantitative proteomics. Age, gender, history of smoking or drinking, and stage of lung cancer did not differ significantly among LUAD, LUSC, and healthy controls (**Supplementary Table S1**). The trends in proteins expression were shown in **Figure 1**. The volcano plot revealed 32 proteins with significant differences between the LUAD and the normal controls, of which 28 proteins were increased and 4 proteins were decreased (*P*<0.05). Moreover, 19 proteins with significant differences (comprising 11 upregulated and 8 downregulated proteins, *P*<0.05) were identified between the LUSC and the normal controls. The detailed results were presented in **Supplementary Tables S2** and **S3**.

### 3.2 Screening of candidate protein markers in early-stage lung cancer by conjoint analysis of proteome and transcriptome

**Figure 2:**
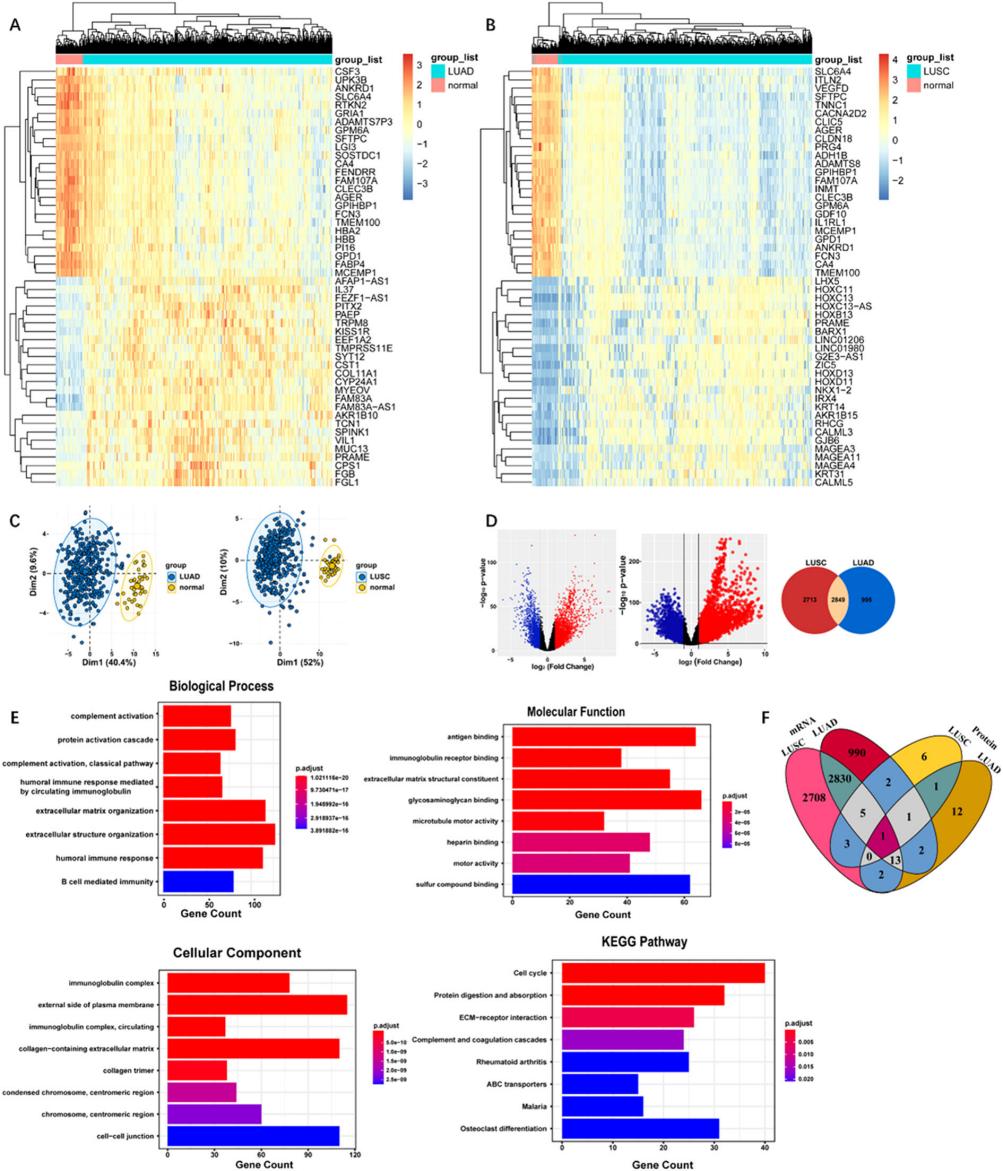
**(A)** Heatmap of DEGs in TCGA LUAD dataset. **(B)** Heatmap of DEGs in TCGA LUSC dataset. **(C)** Principal component analysis of LUAD and LUSC based on the expression levels of the top 100 ranked genes. From left to right: LUAD, LUSC. **(D)** Volcano plot for differentially expressed genes. From left to right: gene expression was compared between LUAD and paracancer tissues, gene expression was compared between LUSC and paracancer tissues, Venn diagram demonstrated the intersections of genes between TCGA LUSC data and TCGA LUAD data. **(E)** The GO and KEGG pathway analysis of the DEGs **(F)** Venn diagram of DEGs shared by plasma and lung cancer tissues.

The clinical information for the TCGA LUAD and LUSC dataset were provided in **Supplementary Tables S4** and **S5**. By conducting clustering of the top 100 ranked genes of LUAD and LUSC, subgroups of tumors with various gene expression patterns were clearly observed (**Figures 2A, B**). PCA suggested that LUAD and normal tissues, as well as LUSC and normal tissues could be effectively classified into two components based on the expression of the top 100 ranked genes, displaying the independence of each group (**Figure 2C**). **Figure 2D** showed dysregulated genes comparing LUAD and paracancer tissues, as well as dysregulated genes comparing LUSC and paracancer tissues. The dysregulated genes that LUAD and LUSC shared were 2849. The GO and KEGG pathway analysis based on DEGs shared by LUAD and LUSC were presented in **Figure 2E**. The top-ranked biological process, molecular function, cellular component, and KEGG pathway were extracellular structure organization, glycosaminoglycan binding, external side of plasma membrane, and cell cycle, respectively. As shown in **Figure 2F** a total of 14 genes (CETP, CAT, LCP1, HYDIN, IGKC, IGLC3, IGHG2, IGHM, IGHV6-1, IGHV3-73, SEPP1, IGKV3-15, IGHV4-34, and ORM1) exhibited differential expressions in the plasma of LUAD and the area of the lesion in lung cancer (including LUAD and LUSC). A total of 6 genes (LYVE1, CLEC3B, FGA, AOC3, HYDIN, and HBB) exhibited differential expressions in the plasma of LUSC and the area of the lesion in lung cancer (including LUAD and LUSC).

### 3.3 Effects of candidate protein markers on the survival of early-stage lung cancer

**Figure 3:**
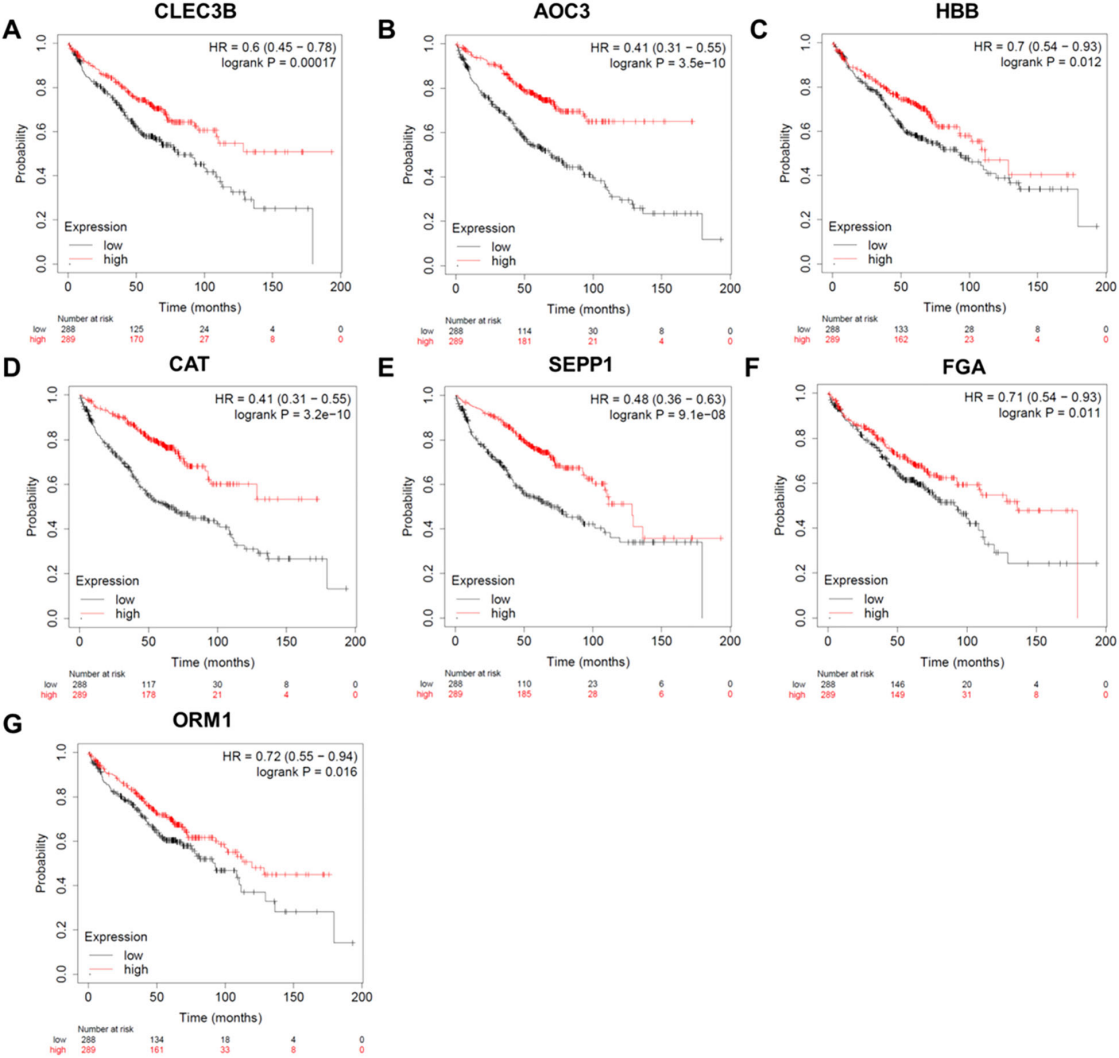
Survival analysis of the candidate protein markers. **(A)** The survival curve of CLEC3B. **(B)** The survival curve of AOC3. **(C)** The survival curve of HBB. **(D)** The survival curve of CAT. **(E)** The survival curve of SEPP1. **(F)** The survival curve of FGA. **(G)** The survival curve of ORM1. HR, hazard ratio; CLEC3B, C-type lectin domain family 3 member B; AOC3, membrane primary amine oxidase; HBB, hemoglobin subunit beta; CAT, catalase; SEPP1, selenoprotein P; FGA, Fibrinogen alpha chain; ORM1, Alpha-1-acid glycoprotein 1.

The survival analysis comprised of 652 individuals with stage I lung cancer. As presented in **Figure 3**, there was a strong correlation between the survival time of individuals with lung cancer and 7 genes (CLEC3B, AOC3, HBB, CAT, SEPP1, FGA, and ORM1, *P*<0.05). As the expression levels of these markers decreased, the total survival probability was significantly reduced.

### 3.4 Verification of candidate protein markers in lung cancer based on microarray technology

**Figure 4:**
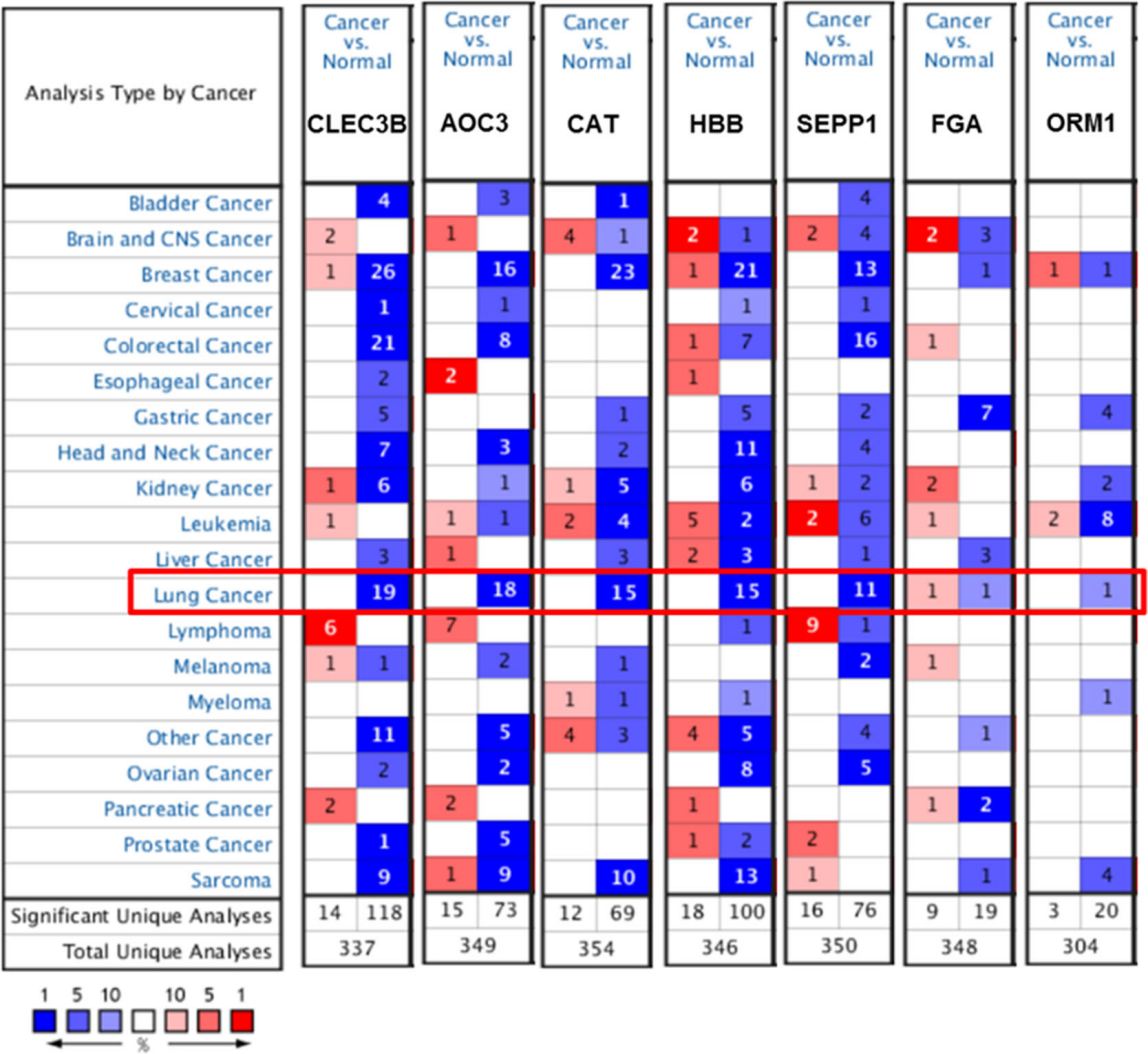
Expression of candidate protein markers in different types of cancer in the Oncomine database.

**Figure 4** illustrated that the expression levels of CLEC3B, AOC3, CAT, HBB and SEPP1 were remarkably downregulated in lung cancer in multiple chips. However, the expression level of FGA was not consistent in the two chips, and the expression level of ORM1 was downregulated in only one chip.

### 3.5 Component identification of coal tar pitch extracts

**Figure 5:**
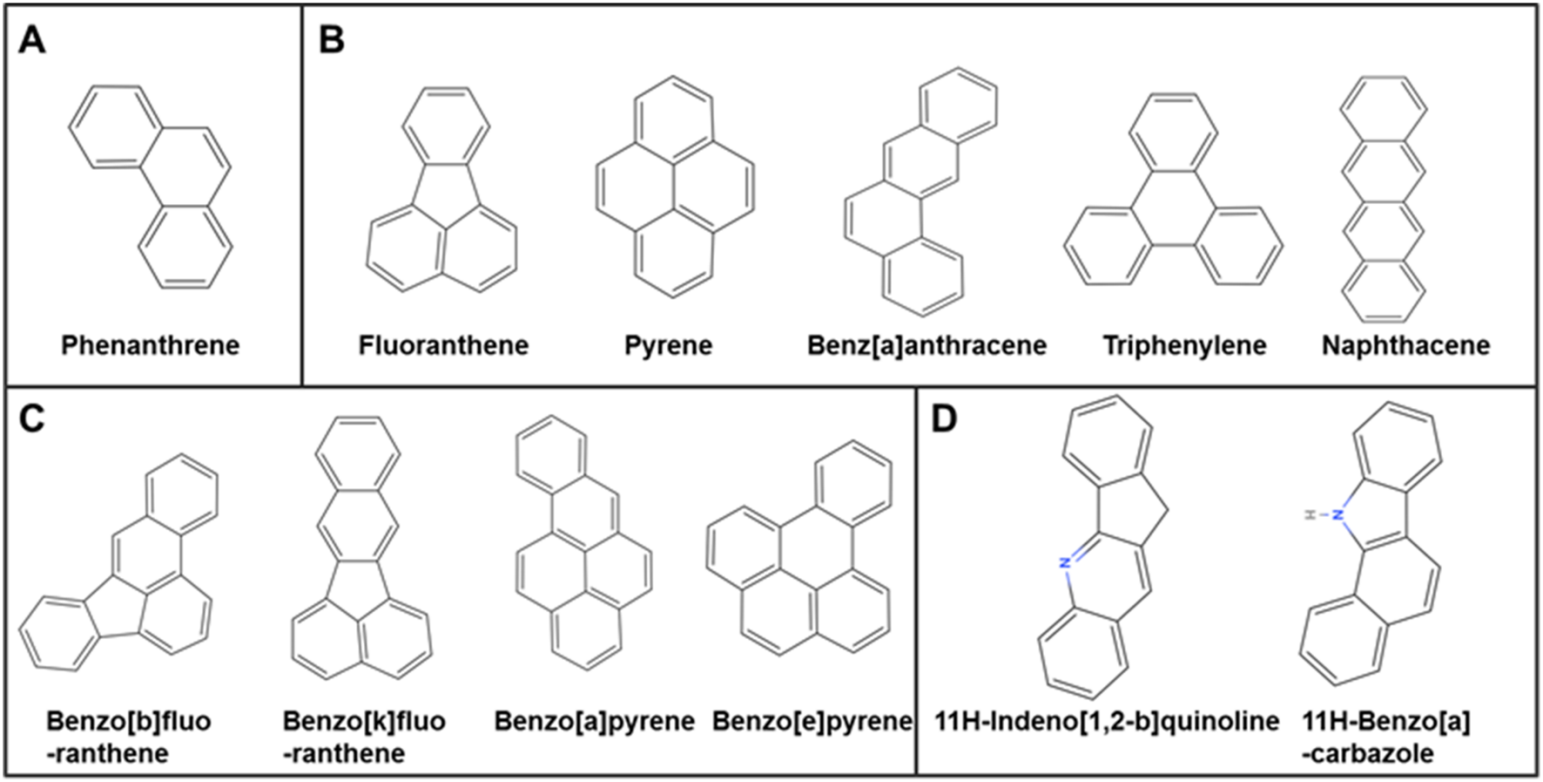
Structural formula of the main compounds of CTPE.

CTPE is rich in polycyclic aromatic hydrocarbons (PAHs) and heterocyclic compounds, representing one of the key carcinogenic constituents in coke oven emissions. Systematic characterization of CTPE components enables a more precise simulation of the chemical exposure profiles encountered by coke oven workers. A total of 34 main compounds were identified in CTPE (**Supplementary Table S6**), including 15 kinds of PAHs (accounting for 43.985% of the total compounds) and 19 kinds of heterocyclic hydrocarbons (accounting for 47.216% of the total compounds). The PAHs mainly consisted of tricyclic, tetracyclic, and pentacyclic aromatic hydrocarbons. As illustrated in **Figure 5**, the tricyclic aromatic hydrocarbons mainly consisted of phenanthrene. The main ingredients of the tetracyclic aromatic hydrocarbons included fluoranthene, pyrene, benz[a]anthracene, triphenylene, and naphthacene. The pentacyclic aromatic hydrocarbons mainly composed of benzo[b]fluoranthene, benzo[k]fluoranthene, benzo[a]pyrene and benzo[e]pyrene. The main ingredients of the heterocyclic compounds included 11H-indeno[1,2-b]quinoline and 11H-benzo[a]carbazole. In the following experiments, *in vitro* and *in vivo* exposure models based on CTPE can facilitate an in-depth investigation of the molecular links between environmental carcinogen exposure and lung cancer development, thereby aiding in the identification of potential early-stage biomarkers for lung cancer.

### 3.6 Development of an *in vitro* model of lung cancer and validation of candidate molecular biomarkers

#### 3.6.1 Carcinogenesis in BEAS-2B cell exposure to CTPE and tumorigenesis in nude mice

**Figure 6:**
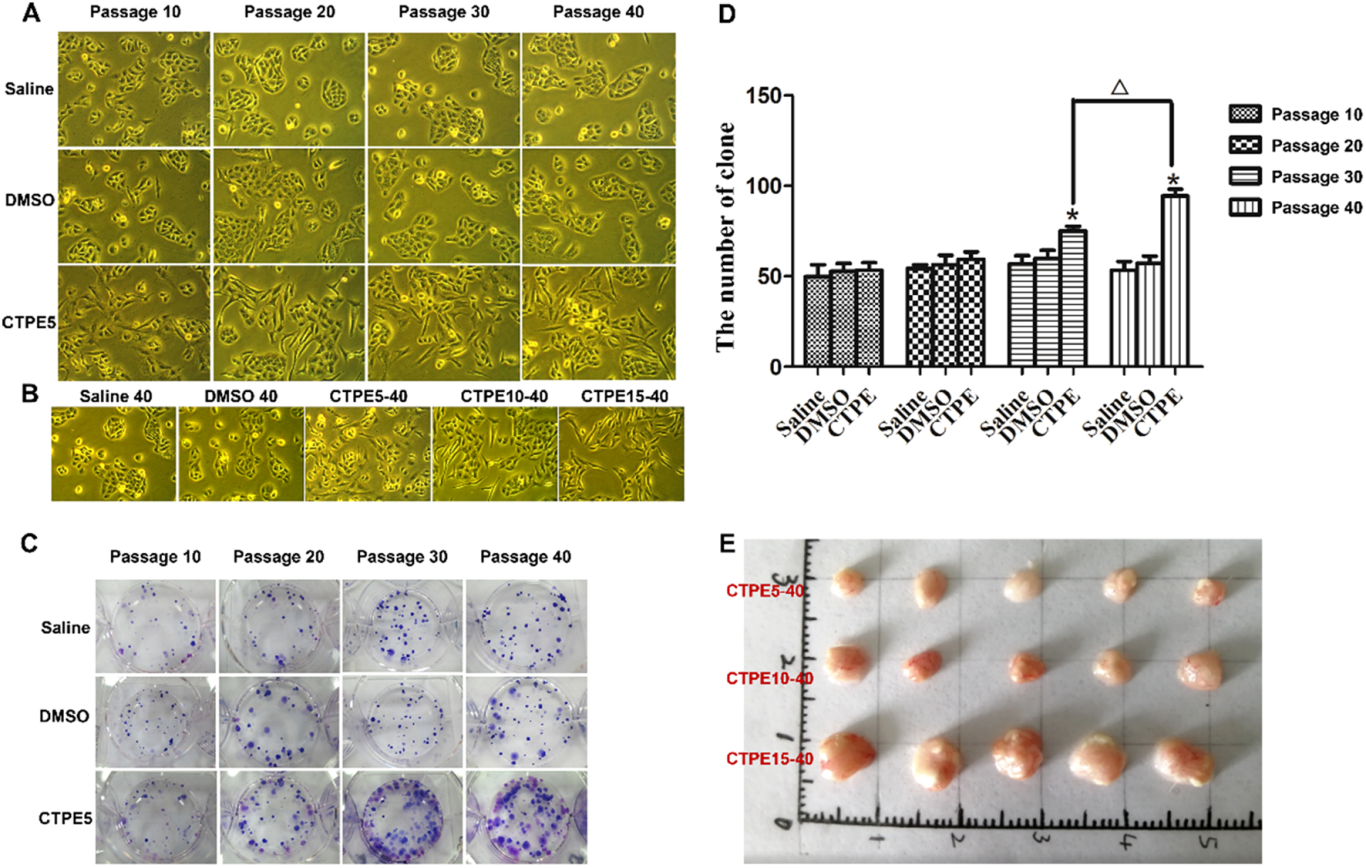
Plate clone formation and tumor formation in nude mice. **(A, B)** Morphological changes of cells in various passages and groups (light micrograph magnifications of 100×). **(C)** Typical clones in various passages and groups. **(D)** The amount of clones in various passages and groups (*n* = 3, *CTPE *vs*. Saline control, *P* < 0.05. △ CTPE5-40 *vs*. CTPE5-30, *P* < 0.05). **(E)** Representatives of tumor removed from nude mice on the 30th day following the injection of BEAS-2B cells induced by CTPE at passage 40.

As depicted in **Figures 6A, B**, the cells in the DMSO group showed no discernible alterations in morphology, while the cells in the CTPE group showed unclear contour, irregular morphology, burr-like changes and vacuole formation in comparison to the saline group. Plate clone formation assay was utilized to assess BEAS-2B cells’ capacity for anchorage-independent growth. As displayed in **Figures 6C, D**, the number of clones of BEAS-2B cells treated with CTPE did not statistically change compared to those of the Saline or DMSO groups at passages 10 and 20 (*P* > 0.05). But at passages 30 and 40, there was a clear increase in the number of clones in the CTPE group in contrast to the saline group (*P* < 0.05). Then, we conducted a tumorigenesis experiment in the nude mice to further verify the malignancy of BEAS-2B cells treated with CTPE at different passages. Clearly, tumors were only formed in nude mice injected with BEAS-2B cells induced by CTPE at passage 40 (**Figure 6E**). These findings showed that the model of malignant transformation of CTPE-induced cells has been established.

#### 3.6.2 Expression of candidate protein markers during the CTPE-induced malignant transformation of BEAS-2B cells

**Figure 7:**
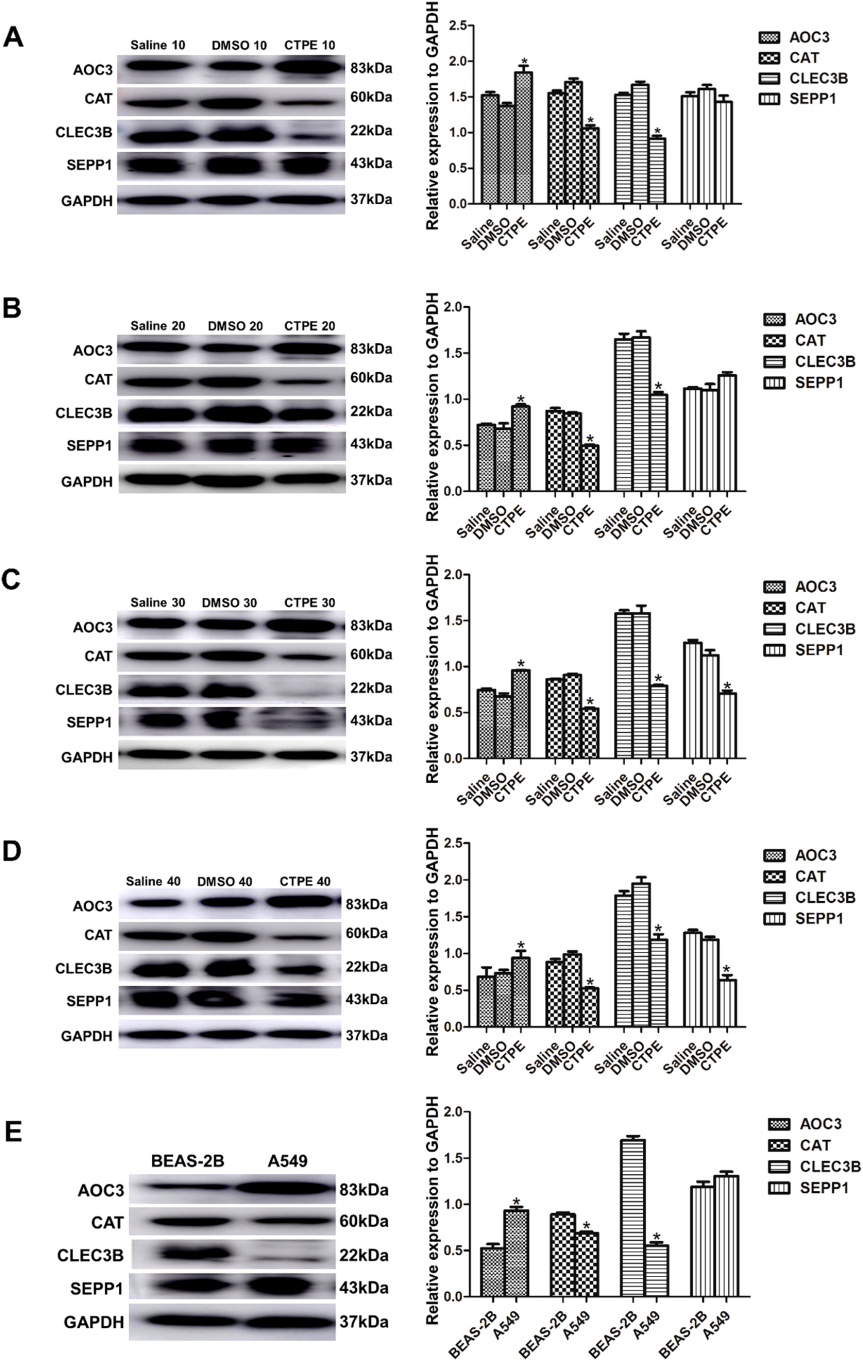
Expression of candidate protein markers in cells of different passages. Expression and comparison of AOC3, CAT, CLEC3B, and SEPP1 among saline group, DMSO group and CTPE exposure group **(A)** in passage 10 (* CTPE *vs*. Saline group, *P* < 0.05). **(B)** in passage 20 (* CTPE *vs*. Saline group, *P* < 0.05). **(C)** in passage 30 (* CTPE *vs*. Saline group, *P* < 0.05). **(D)** in passage 40 (* CTPE *vs*. Saline group, *P* < 0.05). **(E)** Expression and comparison of AOC3, CAT, CLEC3B, and SEPP1 between BEAS-2B and A549 (*: A549 *vs*. BEAS-2B, *P* < 0.05).

As presented in **Figure 7**, the expression of AOC3 was upregulated, while CAT and CLEC3B were downregulated from the 10th passage in the CTPE group compared to the saline group (*P*<0.05). There was no discernible variation in the expression of SEPP1 at passage 10 and passage 20, but the expression of SEPP1 decreased at passage 30 and passage 40 in CTPE-exposed group (*P*<0.05). Meanwhile, the expressions of the four proteins did not differ significantly between the saline and DMSO groups (*P*>0.05). **Figure 7E** reflected that the expression of AOC3 was greater in A549 cells compared to BEAS-2B cells (*P*<0.05). However, A549 cells had lower CAT and CLEC3B expressions than BEAS-2B cells (*P*<0.05). Additionally, the levels of SEPP1 did not differ significantly in A549 cells versus BEAS-2B cells (*P*>0.05).

### 3.7 Development of an *in vivo* model of lung cancer and validation of candidate molecular biomarkers

#### 3.7.1 CTPE-induced tumorigenesis in the lungs of mice

**Figure 8:**
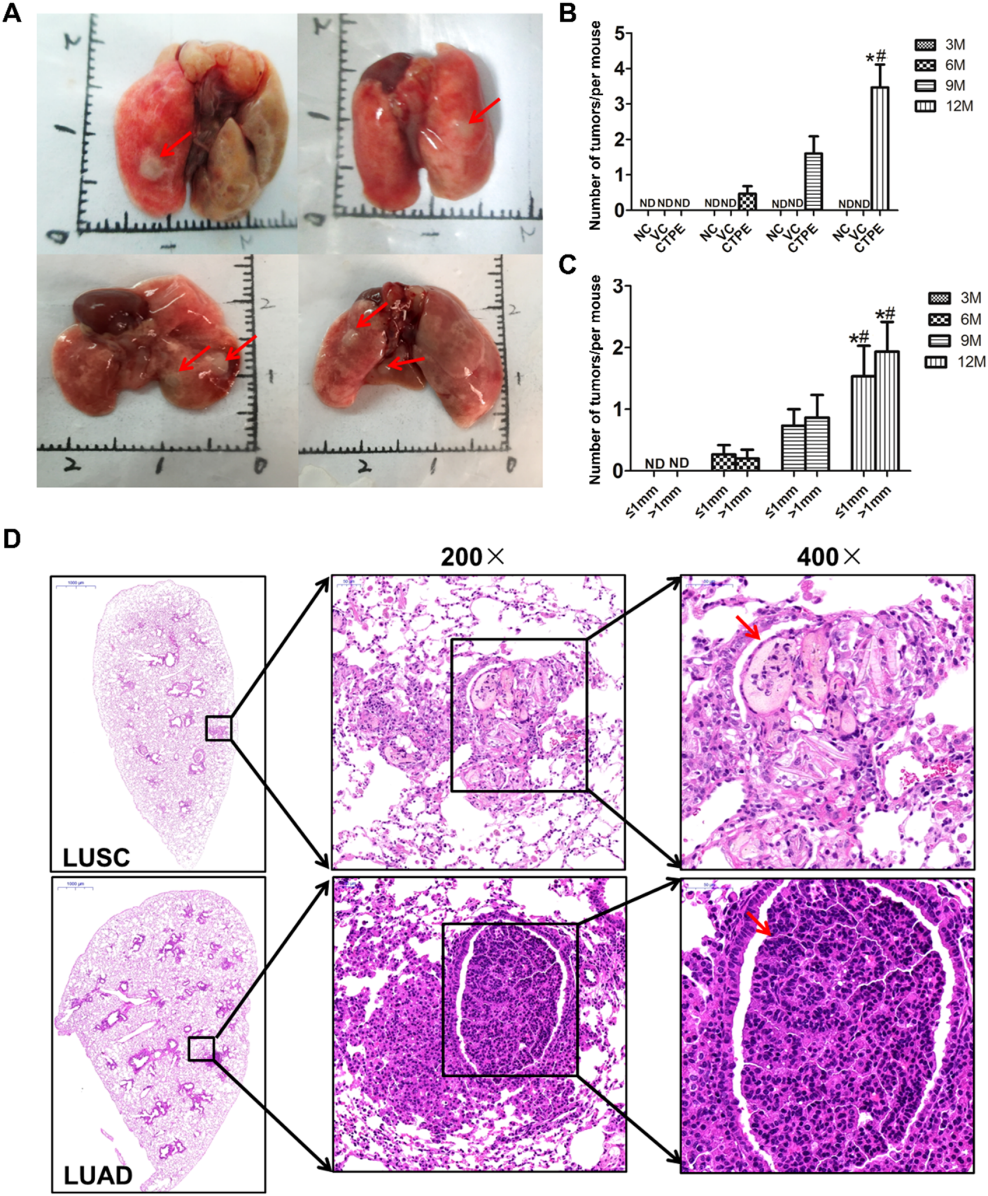
Tumor formation in different groups of mice at different stages. **(A)** General view of the tumors, red arrows indicate the tumors. **(B)** Comparison of the number of tumor formation at different stages in each mice group. **(C)** Comparison of tumor size at different stages of CTPE group. **(D)** Pathological types of CTPE-induced lung carcinogenesis in mice. (Number of tumors, total number of tumors in the lungs of each group of mice/total number of mice in that group; NC, normal control; VC, vehicle control; ND, not detected; * *vs* 6 month, *P* < 0.05; # *vs* 9 month, *P* < 0.05).

Tumors in the lungs of the mice were observed to occur at the 6th, 9th and 12th month after CTPE treatment, most of them had multiple tumors and a few of them had single tumor, which were round, grayish-white and translucent with clear boundaries with the surrounding tissues (**Figure 8A**). **Figure 8B** showed that no tumor was observed in the lungs of the normal control and vehicle control groups at any stage. However, tumors were observed to occur in the lungs of the CTPE group at the 6th, 9th, and 12th month, and the number of tumors at the12th month was higher than that of the 6th and 9th month (*P* < 0.05). **Figure 8C** demonstrated that tumors were of variable sizes and less than 4 mm in diameter, with a higher number of tumors ≤1 mm or >1 mm in diameter at the 12th month than that of the 6th and 9th month. Pathological results indicated that the main types of tumors in the lungs of the mice were LUAD and LUSC (**Figure 8D**).

#### 3.7.2 Expression of candidate protein markers in the lung tissue during lung carcinogenesis in mice induced by CTPE

**Figure 9:**
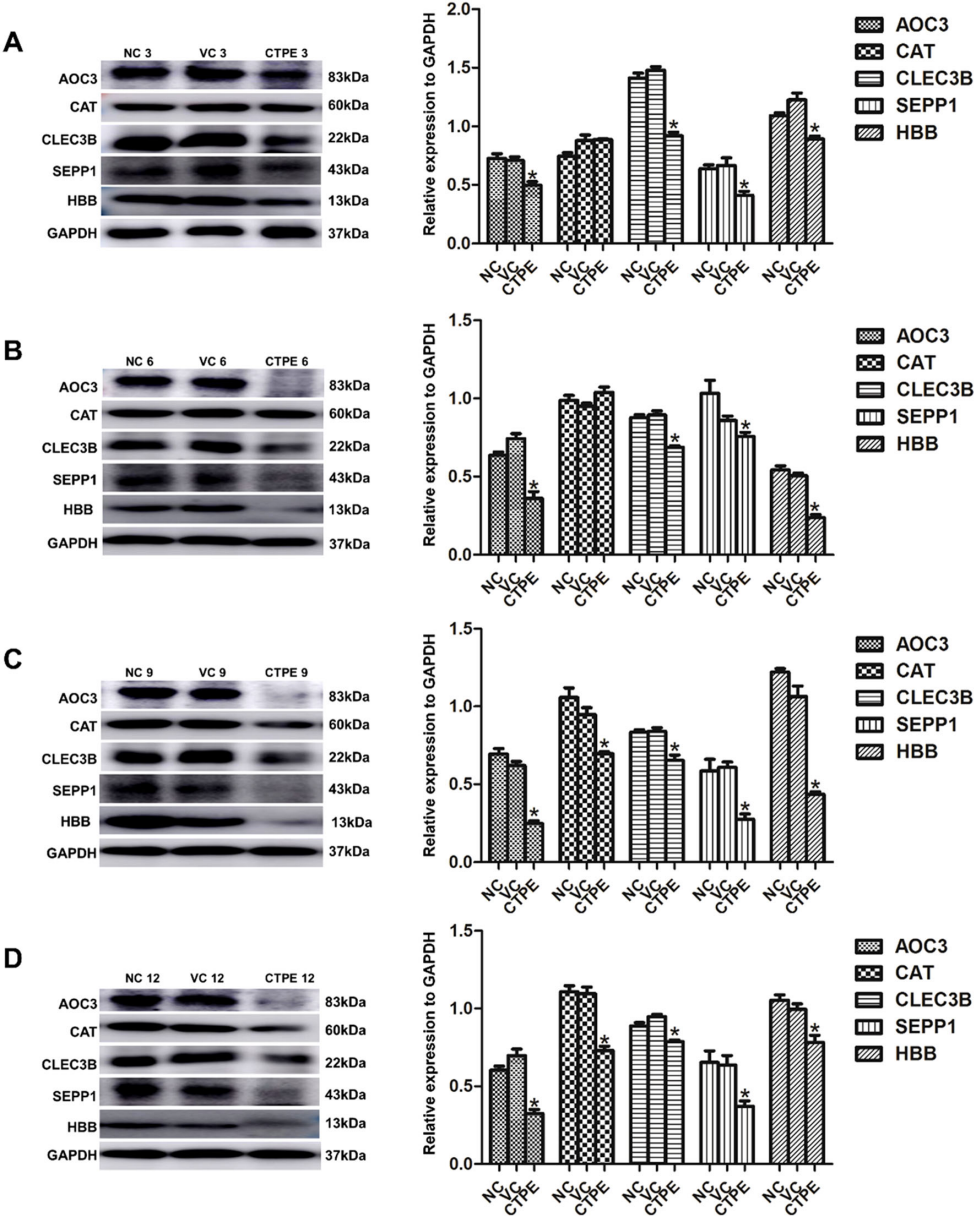
Expression of candidate protein markers in lung tissue of mice in different months. Expressions and comparisons of AOC3, CAT, CLEC3B, SEPP1, and HBB among normal control (NC) group, vehicle control (VC) group and CTPE exposure group **(A)** in the 3rd month. **(B)** in the 6th month. **(C)** in the 9th month. **(D)** in the 12th month (* CTPE *vs*. NC, *P* < 0.05).

As displayed in **Figure 9**, the expressions of AOC3, CLEC3B, SEPP1, and HBB were downregulated (*P*<0.05) in CTPE-treated mice at the 3rd, 6th, 9th, and 12th month in comparison to the normal control group. Moreover, there was no discernible variation in the levels of CAT at the 3rd and 6th month (*P*>0.05), but the expression of CAT decreased at the 9th and 12th month (*P*<0.05). Furthermore, the expressions of the five proteins did not differ between the vehicle control and the normal control in any of the months (*P*>0.05).

#### 3.7.3 Expressions of candidate protein markers in the plasma during the lung carcinogenesis of CTPE-exposed mice

As illustrated in **Table 1**, the expression of AOC3 was elevated in the plasma of CTPE-exposed mice. At the 3rd month, the level of AOC3 in the plasma of mice exposed to CTPE was not different from that of the normal control group (*P*>0.05), while the levels of AOC3 were upregulated in the plasma of CTPE-exposed mice at the 6th, 9th, and 12th month (*P*<0.05). Also, the levels of AOC3 in CTPE-exposed mice at the 12th and 9th month were higher than those at the 3rd month (*P*<0.05). After exposure to CTPE, the expression of CAT initially increased and then decreased. The levels of CAT were higher at the 3rd and 6th month (*P*<0.05) in contrast to the normal control group, while the level of CAT was not different at the 9th month (*P*>0.05) but decreased at the 12th month (*P*<0.05). Additionally, the levels of CAT at the 9th and 12th month were downregulated compared with mice at the 3rd month (*P*<0.05). Moreover, there were no obvious variations in the levels of CLEC3B, SEPP1, and HBB at different time points (*P >*0.05).

**Table 1:** Expression of candidate protein markers in the plasma of mice.

Groups	AOC3 (ng/ml)	CAT (μg/ml)	CLEC3B (μg/ml)	SEPP1 (μg/ml)	HBB (ng/ml)
NC	2.182 ± 0.934	23.24 ± 2.496	0.5503 ± 0.042	0.244 ± 0.181	19.40 ± 0.836
VC	1.455 ± 0.511	18.89 ± 2.020	0.556 ± 0.031	0.227 ± 0.132	18.97 ± 1.376
CTPE3	3.630 ± 0.414	37.07 ± 3.485^*^	0.552 ± 0.105	0.369 ± 0.085	20.03 ± 1.191
CTPE6	5.602 ± 0.963^*^	31.39 ± 2.044^*^	0.578 ± 0.101	0.441 ± 0.160	22.05 ± 0.850
CTPE9	8.053 ± 1.639^*#^	20.63 ± 0.365^#^	0.530 ± 0.028	0.231 ± 0.132	19.49 ± 1.691
CTPE12	9.925 ± 2.074^*#^	12.11 ± 1.433^*#^	0.484 ± 0.033	0.219 ± 0.0159	20.79 ± 2.258
*F*	8.067	17.94	0.243	0.414	0.768
*P*	<0.001	<0.001	0.938	0.830	0.581

* *vs* NC, *P*<0.05; # *vs* CTPE3, *P*<0.05.

#### 3.7.4 Expressions of candidate protein markers in the alveolar lavage fluid during the lung carcinogenesis of CTPE-exposed mice

As shown in **Table 2**, the expression of AOC3 in the alveolar lavage fluid decreased at the 6th and 9th month, and increased at the 12th month (*P*<0.05) after exposure to CTPE in contrast to the normal control group. Additionally, the expression of CAT in the alveolar lavage fluid increased at the 3rd month of CTPE exposure as compared with the normal control group (*P*<0.05), while there were no variations at the 6th, 9th, and 12th month (*P*>0.05). Also, the expression of CLEC3B in the alveolar lavage fluid was decreased at the 6th, 9th, and 12th month (*P*<0.05), and the expression of HBB was decreased at different time points after CTPE exposure (*P*<0.05). Moreover, the levels of SEPP1 at various intervals following CTPE exposure were not substantially different from one another (*P*>0.05). Furthermore, there was no discernible variation in the levels of AOC3, CAT, CLEC3B, SEPP1, and HBB in the alveolar lavage fluid of normal control and vehicle control mice (*P*>0.05).

**Table 2:** Expression of candidate protein markers in the alveolar lavage fluid of mice.

Groups	AOC3 (ng/ml)	CAT (ng/ml)	CLEC3B (ng/ml)	SEPP1 (μg/ml)	HBB (ng/ml)
NC	8.782 ± 0.537	1.863 ± 0.332	7.427 ± 0.889	2.990 ± 0.724	22.70 ± 0.489
VC	8.157 ± 0.537	3.495 ± 0.744	7.195 ± 0.375	3.152 ± 0.343	23.04 ± 0.271
CTPE3	7.419 ± 0.798	18.11 ± 0.895^*^	4.764 ± 0.878	2.515 ± 0.130	19.82 ± 0.843^*^
CTPE6	4.843 ± 0.400^*^	2.837 ± 0.293	4.739 ± 0.336^*^	2.772 ± 0.833	20.21 ± 0.767^*^
CTPE9	4.121 ± 0.319^*^	5.445 ± 1.619	4.392 ± 0.974^*^	3.052 ± 0.476	20.18 ± 0.727^*^
CTPE12	13.60 ± 1.568^*^	5.114 ± 1.409	4.682 ± 0.310^*^	3.061 ± 0.849	20.48 ± 0.855^*^
*F*	6.178	35.27	5.996	0.143	4.380
*P*	0.002	<0.001	0.002	0.979	0.004

* *vs* NC, *P* < 0.05.

### 3.8 Development of a lung cancer screening model and its application in identifying high-risk individuals among coke oven workers

#### 3.8.1 Demographic characteristics and plasma levels of AOC3, CAT, CLEC3B, SEPP1, HBB, CEA, CYFRA21-1, and NSE

There were notable variations in age, sex, smoking history, drinking history, AOC3, CAT, CLEC3B, SEPP1, HBB, CEA, CYFRA21-1, and NSE among the healthy control group, lung cancer group and coke oven workers (*P*<0.05, **Table 3**).

**Table 3:** Demographic characteristics and tumor markers of healthy controls, lung cancer group and coke oven worker group.

Variables	Healthy controls (n=163)	Lung cancer (n=185)	Coke oven worker (n=163)	*χ^2^* / *F*	*P*
Age (year)	75.83 ± 7.21	62.12 ± 10.34	43.76 ± 4.37	686.60	<0.001^*^
Sex (%)					
Men	71 (43.56)	134 (72.43)	136 (83.44)	62.62	<0.001^*^
Women	92 (56.44)	51 (27.57)	27 (16.56)		
Smoking history (%)					
Yes	17 (10.43)	78 (42.16)	80 (49.08)	62.14	<0.001^*^
No	146 (89.57)	107 (57.84)	83 (50.92)		
Drinking history (%)					
Yes	13 (7.98)	48 (25.95)	66 (40.49)	102.39	<0.001^*^
No	150 (92.02)	137 (74.05)	97 (59.51)		
AOC3 (ng/ml)	7.44 (5.91,10.12)	9.27 (7.10,12.92)	7.14 (4.63,10.36)	34.340	<0.001^*^
CAT (ng/ml)	165.9 (103.4,268.7)	173.9 (95.86,309.9)	88.64 (44.07,147.5)	66.083	<0.001^*^
CLEC3B (μg/ml)	0.97 (0.74,1.31)	0.95 (0.68,1.21)	1.06 (0.77,1.45)	9.317	0.009^*^
SEPP1 (μg/ml)	4.54 (3.01,7.06)	5.19 (2.77,8.43)	2.49 (1.59,3.92)	75.990	<0.001^*^
HBB (μg/ml)	108.4 (65.81,222.9)	96.23 (59.24,154.9)	89.57 (53.92,149.6)	12.832	0.002^*^
CEA (ng/ml)	1.47 (1.26,2.09)	2.12 (1.38,9.02)	1.41 (1.23,1.95)	50.737	<0.001^*^
CYFRA21-1 (ng/ml)	0.89 (0.75,1.23)	1.16 (0.87,1.71)	1.02 (0.81,1.39)	21.065	<0.001^*^
NSE (ng/ml)	6.33 (4.29,8.82)	7.31 (4.61,11.13)	5.08 (3.94,9.082)	8.389	0.015^*^

Expressions of proteins were indicated by median (P25, P75).

^*^Statistically significant at *P*=0.05 level.

#### 3.8.2 Effect assessment of machine learning models

As displayed in **Table 4**, the C5.0-8 and ANN-8 models had better performance with AUCs of 0.868(95%*CI*: 0.784-0.928) and 0.844(95%*CI*: 0.756-0.909), respectively. The accuracy, sensitivity, specificity, positive predictive value (PPV), and negative predictive value (NPV) of the C5.0-8 model were 85.57%, 81.97%, 91.67%, 94.34%, and 75.00%, respectively. The corresponding values for the ANN-8 model were 82.47%, 77.05%, 91.67%, 94.00%, and 70.21%, respectively. The five-fold cross-validation results in **Table 5** showed that the average accuracy of the C5.0-8 model was 85.57%, while that of the ANN-8 model was 82.47%.

**Table 4:** Effect assessment of machine learning models in the testing set.

Models	Accuracy (%)	Sensitivity (%)	Specificity (%)	PPV (%)	NPV (%)	AUC (95%*CI*)
C5.0-5	70.10	96.72	25.00	68.60	81.82	0.609 (0.504-0.706)
ANN-5	76.29	78.69	72.22	82.76	66.67	0.755 (0.657-0.836)
SVM-5	68.04	91.80	27.78	68.29	66.67	0.598 (0.493-0.696)
Fisher-5	73.20	78.69	63.89	78.69	63.89	0.713 (0.612-0.800)
C5.0-3	65.98	60.66	75.00	80.43	52.94	0.678 (0.576-0.770)
ANN-3	67.01	67.21	66.67	77.36	54.55	0.669 (0.567-0.762)
SVM-3	60.82	49.18	80.56	81.08	48.33	0.649 (0.545-0.743)
Fisher-3	56.70	40.98	83.33	80.65	45.45	0.622 (0.517-0.718)
C5.0-8	85.57	81.97	91.67	94.34	75.00	0.868 (0.784-0.928)
ANN-8	82.47	77.05	91.67	94.00	70.21	0.844 (0.756-0.909)
SVM-8	68.04	60.66	80.56	84.09	54.72	0.706 (0.605-0.794)
Fisher-8	75.26	70.49	83.33	87.76	62.50	0.769 (0.673-0.849)

**Table 5:** Five-fold cross-validation results for the C5.0-8 and ANN-8 models.

Models	Accuracy (%)	Sensitivity (%)	Specificity (%)	PPV (%)	NPV (%)	AUC (95%*CI*)
K1-C5.0	85.57	81.97	91.67	94.34	75.00	0.868 (0.784-0.928)
K2-C5.0	85.57	80.33	94.44	96.08	73.91	0.874 (0.811-0.937)
K3-C5.0	84.54	78.69	94.44	96.00	72.34	0.866 (0.801-0.930)
K4-C5.0	85.57	83.61	88.89	92.73	76.19	0.862 (0.792-0.933)
K5-C5.0	86.60	85.25	88.89	92.86	78.05	0.871 (0.802-0.939)
K1-ANN	82.47	77.05	91.67	94.00	70.21	0.844 (0.756-0.909)
K2-ANN	82.47	75.41	94.44	95.83	69.39	0.849 (0.783-0.916)
K3-ANN	81.44	73.77	94.44	95.74	68.00	0.841 (0.774-0.908)
K4-ANN	82.47	78.69	88.89	92.31	71.11	0.838 (0.764-0.911)
K5-ANN	83.51	80.33	88.89	92.45	72.73	0.846 (0.774-0.918)

#### 3.8.3 Prediction of high-risk individuals of lung cancer in coke oven workers by the optimal models

The C5.0-8 and ANN-8 models were combined to predict the high-risk individuals of lung cancer among coke oven workers, and a total of 14 high-risk individuals were screened. The baseline features of high-risk individuals were described in **Table 6**. Subjects had mostly experienced long-term occupational exposure, and 9 high-risk individuals had been working for more than 25 years. In addition, 8 high-risk individuals had a long-term smoking history.

**Table 6:** Basic information of 14 high-risk individuals.

Number	Gender	Age (years)	Smoking history	Type of work	Occupational exposure (years)
406	Male	46	15 years, 15/day	Uplift pipefitter	25
461	Male	46	12 years, 20/day	Furnace door operator	28
465	Male	51	14 years, 20/day	Uplift pipefitter	29
576	Male	55	20 years, 20/day	Furnace cover worker	35
668	Male	48	No	Coke-blocker driver	21
682	Male	45	No	Coke-blocker driver	27
694	Male	46	No	Furnace door repairer/Coke quenching worker	28
740	Male	47	22 years, 10/day	Coal truck driver	28
786	Male	43	20 years, 20/day	hot repairer	28
795	Male	40	no	Coke-blocker driver	22
805	Male	49	20 years, 15/day	Furnace door operator	15
843	Male	38	12 years, 20/day	Coke-extinguishing truck driver	15
861	Male	48	No	Furnace door operator	14
877	Male	47	No	Coke pusher driver	28

## 4 Discussion

The search for highly sensitive and specific biomarkers of early-stage lung cancer is beneficial in reducing the burden of lung cancer on society. In this study, we performed a series of bioinformatics analysis on the differentially expressed proteins and genes in early-stage lung cancer. The early candidate protein markers of lung cancer in peripheral blood were screened by label-free quantitative proteomics. The candidate protein markers were investigated based on the TCGA database, supplemented by Kaplan-Meier plotter and Oncomine analysis, and then AOC3, CLEC3B, CAT, SEPP1, and HBB were selected as potential candidates for early-stage lung cancer. The *in vivo* and *in vitro* models of lung carcinogenesis were successfully constructed by inducing malignant transformation of BEAS-2B cells and lung carcinogenesis by CTPE in C57BL/6 mice. The expressions of candidate protein markers were dynamically observed at the various phases of lung carcinogenesis, which revealed that AOC3, CAT, CLEC3B, SEPP1, and HBB may be the key molecular markers of early lung cancer lesions. Finally, the screening models for lung cancer were constructed by combining candidate protein markers (AOC3, CAT, CLEC3B, SEPP1, HBB) with tumor markers (CEA, CYFRA21-1, NSE) using machine learning. The decision tree C5.0 and ANN models had better performance, and a total of 14 high-risk individuals were screened among coke oven workers.

CTPE is a confirmed human carcinogen and consists mostly of PAHs and heterocyclic compounds by GC/MS analysis. Occupational exposure of coke oven workers to PAHs has been associated with lung cancer and other adverse health effects (26). Tumorigenesis in immunodeficient mice is the gold standard for detecting malignant transformation of BEAS-2B cells (27). **Figure 6** illustrated that the CTPE-induced malignant transformation model of BEAS-2B cells was effectively established. This study further examined the cancerous nature of BEAS-2B cells under various modes of CTPE treatment. It was found that an increase in the number of CTPE treatment can enhance the cancerous nature of BEAS-2B cells. Epidemiological studies (28) among coke oven workers showed that lung cancer risk is not only related to the exposure dose of carcinogens in the work environment, but also closely related to the length of work, and a longer exposure history has the potential to raise lung cancer risk. Based on the previous study, this study successfully induced lung carcinogenesis in C57BL/6 mice by tracheal drip of 1.0 mg CTPE per each mouse. The main pathological types included LUSC, LUAD and adenosquamous carcinoma. Consistent with this result, lung cancer predominantly consists of LUAD and LUSC (29).

AOC3 is an endothelial adhesion molecule that plays a crucial role in mediating cell adhesion. Moreover, it has been implicated in oxidative stress responses, vascular inflammation, and leukocyte adhesion, processes that are closely associated with the early stages of carcinogenesis. Under normal conditions, AOC3 is highly expressed in smooth muscle cells, vascular endothelial cells and adipocytes (30). The investigation revealed that the CTPE group and A549 cells had higher levels of AOC3 expression. It has been shown that cell migration, adhesion and colony formation are significantly impaired when AOC3 expression is knocked down (31). Based on this work, it is possible that malignant transformation of cells is facilitated by increased expression of AOC3. However, the expression of AOC3 is downregulated in some cancers such as aggressive prostate and colorectal cancers (32, 33). AOC3 has been reported to mediate the adhesion of lymphocytes infiltrating around tumors to various cancerous tissues, killing cancer cells and thus exerting an inhibitory effect on tumors (32). In this study, the downregulation of AOC3 was found in the lung tissue of mice at the 3rd, 6th, 9th, and 12th month after CTPE exposure, which is contrary to the results of the cellular assay described above. It is hypothesized that AOC3 *in vivo* interacts with other substances or cells to interfere with the process of carcinogenesis. In contrast, the cell experiments were conducted in a single environment and the expression of AOC3 was not regulated by feedback from other substances or cells in the *in vivo* environment. Studies (34) have reported that AOC3 can also recruit myeloid cells into tumors to enhance tumor growth. In this study, AOC3 was gradually upregulated in the plasma of CTPE-exposed mice, while it was downregulated in the lavage fluid of mice aged 6 and 9 months and upregulated in the lavage fluid of mice aged 12 months, suggesting that AOC3 may function differently at different sites during lung carcinogenesis, leading to different trends in its expression.

Oxidative stress is a key component in carcinogenesis (35). Altered redox homeostasis has been demonstrated in tumor cells. One of the main redox metabolites, hydrogen peroxide functions as a second messenger and it is essential for cell morphological changes, cell proliferation, and apoptosis (36). CAT is the key enzyme for the elimination of the toxicity of hydrogen peroxide (37). According to this study, the expression of CAT was downregulated in CTPE group and A549 cells. CAT is a critical enzyme for scavenging oxygen radicals. However, a large accumulation of oxygen radicals can cause an imbalance of the antioxidant-oxidant system, resulting in a decline in the production and activity of CAT. In this study, the expression of CAT decreased in the lung tissues of mice at the 9th and 12th month after CTPE exposure. The mice were mostly in the initial stages of lung carcinogenesis at the 9th month after CTPE exposure. It is therefore hypothesized that the initial stages of CTPE-induced lung carcinogenesis may be characterized by an imbalance in the oxidative stress, which in turn leads to the downregulation of CAT. Consistently, it has been documented that CAT is lowly expressed in early-stage lung cancer tissues (38). Furthermore, CAT was significantly upregulated and then gradually downregulated in the plasma of mice induced by CTPE in this study. It was also significantly upregulated in the lavage fluid of mice induced by CTPE at the 3rd month. It is suggested that CTPE exposure triggers a strong oxidative stress response in mice at the early stages of poisoning. CAT is then released to the alveolar surface and blood to regulate the oxidative stress response, and when the antioxidant system is insufficient to scavenge oxygen radicals, the levels of CAT would be downregulated.

CLEC3B is found mainly in the cytoplasm, extracellular matrix, and exosomes. The downregulation of CLEC3B promotes cell migration, invasion and epithelial-mesenchymal transition, and also affects tumor angiogenesis by regulating VEGF expression through the AMPK signaling pathway. The investigation suggested that the expression of CLEC3B was downregulated in CTPE group and A549 cells. In addition, the expression of CLEC3B was downregulated in some tumor tissues, such as renal cell and hepatocellular carcinomas (39, 40). It has been reported that the level of CLEC3B is downregulated in stage IA lung cancer, suggesting that CLEC3B could be a useful biomarker for lung cancer diagnosis and prognosis (41). In this research, CLEC3B was downregulated in the lung tissues of mice at the 3rd, 6th, 9th and 12th month after CTPE exposure. Moreover, the expression of CLEC3B was downregulated in the lavage fluid of mice induced by CTPE at the 6th, 9th, and 12th month. The findings of this study confirmed that CLEC3B is significantly downregulated and may be a key regulator in the early stages of lung carcinogenesis.

Selenium is an essential trace element for humans and animals, and Selenoprotein P (SEPP1) is the major protein for selenium transport (42). SEPP1 serves antioxidant functions and can inhibit oxygen radical-mediated DNA damage, gene mutation, and tumor initiation by scavenging oxygen free radicals (43). In this study, the expression of SEPP1 was downregulated in BEAS-2B cells stimulated by CTPE at passage 30 and passage 40 and the lung tissues of mice at the 3rd, 6th, 9th, and 12th month after CTPE exposure. Previous study had found significant downregulation of SEPP1 expression in tumorous lung tissue. Potentially, downregulating SEPP1 expression could raise oxidative stress and promote the occurrence of lung cancer (44).

Peptide mapping in the beta subunit of HBB is defined as the C-terminal region responsible for activity and is a key vehicle for oxygen transport *in vivo (* 45). Moreover, HBB is an anti-metastatic factor with antitumor effects, inhibiting the proliferation of tumor cells (46, 47). However, the expression of HBB was not detected in either BEAS-2B cells or CTPE-induced BEAS-2B cells. It was speculated that there was very little expression of HBB in these cells and the detection method was not sensitive enough. Thus, more sensitive detection methods will be used for detection in subsequent studies. Following exposure to CTPE, the lung tissues of mice showed a downregulation of HBB expression at the 3rd, 6th, 9th, and 12th month. According to reports, HBB expression in the serum of ovarian cancer patients is higher than in normal controls, suggesting that it could be used as a potential diagnostic marker for the disease (48). Nevertheless, HBB was not differentially expressed in the plasma of mice in this study, but it was significantly downregulated in the lavage fluid, which agrees with the findings of HBB expression in the lung tissue of mice.

Currently, the most popular diagnostic and prognostic markers for lung cancer are CEA, NSE, and CYFRA21-1 (13). Patients with LUAD have higher expression levels of CEA. NSE is used for the differential diagnosis of SCLC and the assessment of treatment outcomes. CYFRA21-1 is a tumor marker in LUSC and is significantly elevated in patients with LUSC. This study confirmed that the expressions of CEA, NSE, and CYFRA21-1 were higher in the plasma of the lung cancer group than those of healthy controls. Additionally, the plasma of coke oven workers showed the substantial upregulation of CYFRA21-1, indicating that exposure to coke oven emission may enhance the risk of LUSC in these workers. Furthermore, the expression of AOC3 was increased in the plasma of individuals with lung cancer, which was consistent with the results in the plasma of mice. Moreover, the expressions of CLEC3B and HBB were reduced in the lung cancer group. In addition, the decreased expressions of CAT and SEPP1 in the coke oven workers suggested that the workers might be in a state of oxidative stress due to long term exposure to coke oven emission.

Machine learning is a data processing method developed based on computer technology and is widely used in the field of medical research. In this study, the decision tree C5.0 and ANN models which combined 8 molecular markers outperformed models based on 5 candidate protein markers (AOC3, CAT, CLEC3B, SEPP1, and HBB) or 3 traditional markers (CEA, NSE, and CYFRA21-1). The C5.0-8 and ANN-8 models were then used to predict high-risk individuals. A total of 14 coke oven workers were screened out with 8 of them having a long history of smoking. Tobacco smoke exposure can increase the risk of lung cancer, which is the main risk factor for lung cancer (49). Research has revealed that smoking can increase the risk of certain diseases in coke oven workers (50, 51). This study revealed that the long history of smoking combined with occupational exposure may increase the risk of lung cancer. Furthermore, only one of the high-risk individuals had changed job types, while the remaining 13 coke oven workers have been working in a single job type for a long time. Lung cancer develops over a lengthy period of time, and long-term exposure to PAHs may lead to persistent oxidative stress in the lung, which in turn induces DNA adduct formation, gene mutations, and chromosomal mutations (7). DNA repair is a key regulatory pathway in the body’s response to exogenous and endogenous damage, and prolonged and sustained exposure to environmental toxicants may impair the DNA repair capacity of the lung, resulting in the inability to reverse DNA damage, leading to the emergence of lung cancer (52). Therefore, regular changes in work types may facilitate the repair of the damage of oxidative stress in the lungs and reduce the risk of lung cancer.

The study aimed to screen for early molecular indicators and to construct screening models of lung cancer, through the use of a population-based study, complemented by animal and cellular experiments, to demonstrate the relevant results from different perspectives and to provide more reliable evidence for the study. However, the study has several shortcomings. Firstly, it lacked studies on the function and mechanism of candidate protein markers in lung carcinogenesis. Secondly, the study had a limited number of lung cancer cases at initial stages, which may reduce the effectiveness of the model in early screening for lung cancer. To validate and optimize the constructed models, we plan to expand the sample size through multi-center studies, particularly samples of lung cancer at initial stages, in a subsequent study. Moreover, *in vivo* and ex vivo experiments will be conducted to investigate the mechanisms and functions of candidate protein markers in the formation of lung cancer and to establish an experimental foundation for lung cancer treatment. Furthermore, a prospective longitudinal follow-up will be conducted for the 14 high-risk coke oven workers to monitor their health status and verify the potential development of lung cancer. Specifically, these individuals will undergo LDCT and biomarker testing every six months as part of the regular follow-up.

## 5 Conclusion

The differentially expressed molecules of lung cancer at initial stages were identified by multi-omics technologies. These molecules were observed and validated using *in vivo* and *in vitro* models as well as population samples, and it was revealed that altered expression of AOC3, CAT, CLEC3B, SEPP1, and HBB proteins were early molecular events in lung carcinogenesis. The candidate protein markers (AOC3, CAT, CLEC3B, SEPP1, HBB) and traditional tumor markers (CEA, CYFRA21-1, NSE) were combined to construct screening models for lung cancer by machine learning techniques. The decision tree C5.0 and ANN had better predictive performance and they could be applied to screen high-risk individuals in coke oven workers. It might provide a new strategy and method for the early detection and diagnosis of lung cancer.

## Data Availability

The original contributions presented in the study are included in the article/**Supplementary Material**. Further inquiries can be directed to the corresponding authors.
