# Supplementary material for: Screening and identification of novel protein markers of early-stage lung cancer and construction and application of screening models
Source: Front Oncol. 2025 May 27;15:1567673. doi: 10.3389/fonc.2025.1567673 (PMC12149181; doi:10.3389/fonc.2025.1567673)
Supplement: Supplementary file 1 [file Table1.docx]

List of Contents

1. Basic characteristics of clinical research subjects

2. Differentially expressed proteins in the peripheral blood of patients with LUAD

3. Differentially expressed proteins in the peripheral blood of patients with LUSC

4. The clinical information and sample size for TCGA LUAD dataset

5. The clinical information and sample size for TCGA LUSC dataset

6. Chemical constituents of the coal tar pitch extracts

1. **Basic characteristics of clinical research subjects**

**Table S1** Basic characteristics of clinical research subjects

| Variables | LUAD | LUSC | Healthy controls | *χ^2^*／*F* | *P* |
| --- | --- | --- | --- | --- | --- |
| Age(years) | 64.60$\pm$6.66 | 64.40±8.85 | 67.88±6.69 | 0.470 | 0.632 |
| Gender |  |  |  |  | 0.176* |
| Male (%) | 4(80.00) | 5(100.00) | 6(75.00) |  |  |
| Female (%) | 1(20.00) | 0(0) | 2(25.00) |  |  |
| Smoking history (%) | 3(60.00) | 4(80.00) | 5(62.50) |  | 0.151* |
| Drinking history (%) | 1(20.00) | 2(40.00) | 1(12.50) |  | 0.131* |
| Lung cancer stage |  |  |  |  | 0.897* |
| Stage I (%) | 3(60.00) | 4(80.00) |  |  |  |
| Stage II (%) | 2(40.00) | 1(20.00) |  |  |  |

*, Fisher's exact test

**2. Differentially expressed proteins in the peripheral blood of patients with LUAD**

**Table S2** Differentially expressed proteins in the peripheral blood of patients with LUAD

| Number  of protein | Protein | Gene | Fold change | *P-*value | Trend |
| --- | --- | --- | --- | --- | --- |
| P02652 | Apolipoprotein A-II | APOA2 | 0.432 | 0.028 | Down |
| P04114 | Apolipoprotein B-100 | APOB | 0.569 | 0.027 | Down |
| P11597 | Cholesteryl ester transfer protein | CETP | 0.602 | 0.004 | Down |
| P04180 | Phosphatidylcholine-sterol acyltransferase | LCAT | 0.650 | 0.017 | Down |
| P00390 | Glutathione reductase，mitochondrial | GSR | 1.568 | 0.026 | Up |
| P08253 | 72 kDa type IV collagenase | MMP2 | 1.576 | 0.001 | Up |
| Q86U17 | Serpin A11 | SERPINA11 | 1.631 | 0.016 | Up |
| Q9UK55 | Protein Z-dependent protease inhibitor | SERPINA10 | 1.633 | 0.041 | Up |
| P09972 | Fructose-bisphosphate aldolase C | ALDOC | 1.648 | 0.002 | Up |
| O43866 | CD5 antigen-like | CD5L | 1.679 | 0.022 | Up |
| Q4G0P3 | Hydrocephalus-inducing protein homolog | HYDIN | 1.866 | 0.007 | Up |
| P22891 | Vitamin K-dependent protein Z | PROZ | 1.905 | 0.021 | Up |
| P49908 | Selenoprotein P | SEPP1 | 2.002 | 0.013 | Up |
| P13796 | Plastin-2 | LCP1 | 2.296 | 0.004 | Up |
| P01859 | Immunoglobulin heavy constant gamma 2 | IGHG2 | 2.631 | 0.002 | Up |
| P01871 | Immunoglobulin heavy constant mu | IGHM | 2.743 | 0.001 | Up |
| A0A0B4J1U7 | Immunoglobulin heavy variable 6-1 | IGHV6-1 | 2.953 | 0.013 | Up |
| P0DOY3 | Immunoglobulin lambda constant 3 | IGLC3 | 3.153 | 0.013 | Up |
| P30043 | Flavin reductase （NADPH） | BLVRB | 3.724 | 0.020 | Up |
| P32119 | Peroxiredoxin-2 | PRDX2 | 3.829 | 0.005 | Up |
| P04040 | Catalase | CAT | 3.835 | 0.040 | Up |
| A0A0B4J1V6 | Immunoglobulin heavy variable 3-73 | IGHV3-73 | 3.932 | 0.007 | Up |
| P01834 | Immunoglobulin kappa constant | IGKC | 3.984 | 0.013 | Up |
| P00915 | Carbonic anhydrase 1 | CA1 | 3.985 | 0.006 | Up |
| P01701 | Immunoglobulin lambda variable 1-51 | IGLV1-51 | 4.114 | 0.037 | Up |
| P01624 | Immunoglobulin kappa variable 3-15 | IGKV3-15 | 4.265 | 0.036 | Up |
| P02763 | Alpha-1-acid glycoprotein 1 | ORM1 | 4.268 | 0.044 | Up |
| A2NJV5 | Immunoglobulin kappa variable 2-29 | IGKV2-29 | 4.569 | 0.001 | Up |
| P01619 | Immunoglobulin kappa variable 3-20 | IGKV3-20 | 4.585 | 0.035 | Up |
| P06331 | Immunoglobulin heavy variable 4-34 | IGHV4-34 | 4.721 | 0.009 | Up |
| P00918 | Carbonic anhydrase 2 | CA2 | 4.916 | 0.004 | Up |
| P69905 | Hemoglobin subunit alpha | HBA1 | 12.298 | 0.031 | Up |

**3. Differentially expressed proteins in the peripheral blood of patients with LUSC**

**Table S3** Differentially expressed proteins in the peripheral blood of patients with LUSC

| Number  of protein | Protein | Gene | Fold change | *P-*value | Trend |
| --- | --- | --- | --- | --- | --- |
| Q9Y5Y7 | Lymphatic vessel endothelial hyaluronic acid receptor 1 | LYVE1 | 0.30 | 0.014 | Down |
| P09172 | Dopamine beta-hydroxylase | DBH | 0.43 | 0.028 | Down |
| O00533 | Neural cell adhesion molecule L1-like protein | CHL1 | 0.54 | 0.020 | Down |
| Q16853 | Membrane primary amine oxidase | AOC3 | 0.57 | 0.038 | Down |
| P05452 | Tetranectin | CLEC3B | 0.59 | 0.033 | Down |
| Q9UNW1 | Multiple inositol polyphosphate phosphatase 1 | MINPP1 | 0.64 | 0.047 | Down |
| Q96PD5 | N-acetylmuramoyl-L-alanine amidase | PGLYRP2 | 0.65 | 0.049 | Down |
| P29622 | Kallistatin | SERPINA4 | 0.66 | 0.007 | Down |
| P02671 | Fibrinogen alpha chain | FGA | 1.69 | 0.007 | Up |
| P02679 | Fibrinogen gamma chain | FGG | 1.77 | 0.013 | Up |
| P02675 | Fibrinogen beta chain | FGB | 1.78 | 0.011 | Up |
| P00918 | Carbonic anhydrase 2 | CA2 | 1.88 | 0.044 | Up |
| P04003 | C4b-binding protein alpha chain | C4BPA | 1.94 | 0.001 | Up |
| Q4G0P3 | Hydrocephalus-inducing protein homolog | HYDIN | 1.95 | 0.017 | Up |
| P01009 | Alpha-1-antitrypsin | SERPINA1 | 2.23 | 0.027 | Up |
| P18428 | Lipopolysaccharide-binding protein | LBP | 2.80 | 0.002 | Up |
| P69905 | Hemoglobin subunit alpha | HBA1 | 6.50 | 0.049 | Up |
| P68871 | Hemoglobin subunit beta | HBB | 7.73 | 0.041 | Up |
| P02741 | C-reactive protein | CRP | 12.25 | 0.042 | Up |

4. **The clinical information and sample size for TCGA LUAD dataset**

**Table S4** The clinical information and sample size for TCGA LUAD dataset

| Variables | LUAD (n=395) | Controls (n=43) | *χ^2^*／*t* | *P* |
| --- | --- | --- | --- | --- |
| Age(years) | 65.52 ± 9.84 | 66.84 ± 10.14 | 0.843 | 0.407 |
| Gender |  |  | 0.030 | 0.863 |
| Male (%) | 180（45.6） | 19（44.2） |  |  |
| Female (%) | 215（54.4） | 24（55.8） |  |  |
| Lung cancer stage |  |  | 0.003 | 0.957 |
| Stage I (%) | 274（69.4） | 30（69.8） |  |  |
| Stage II (%) | 121（30.6） | 13（30.2） |  |  |
| sampling site |  |  | 0.011 | 0.994 |
| Upper lobe of the lung (%) | 241（61.0） | 26（60.5） |  |  |
| Lower lobe of the lung (%) | 128（32.4） | 14（32.6） |  |  |
| Others (%) | 26（6.6） | 3（7.0） |  |  |

5. **The clinical information and sample size for TCGA LUSC dataset**

**Table S5** The clinical information and sample size for TCGA LUSC dataset

| Variables | LUSC (n=406) | Controls (n=43) | *χ^2^*／*t* | *P* |
| --- | --- | --- | --- | --- |
| Age(years) | 67.54 ± 8.72 | 69.14 ± 8.68 | 1.141 | 0.254 |
| Gender |  |  |  |  |
| Male (%) | 297（73.2） | 31（72.1） | 0.022 | 0.882 |
| Female (%) | 109（26.8） | 12（27.9） |  |  |
| Lung cancer stage |  |  | 0.002 | 0.963 |
| Stage I (%) | 244（60.1） | 26（60.5） |  |  |
| Stage II (%) | 162（39.9） | 17（39.5） |  |  |
| sampling site |  |  | 0.289 | 0.866 |
| Upper lobe of the lung (%) | 209（51.5） | 23（53.5） |  |  |
| Lower lobe of the lung (%) | 148（36.5） | 16（37.2） |  |  |
| Others (%) | 49（12.0） | 4（9.3） |  |  |

6. **Chemical constituents of the coal tar pitch extracts**

**Table S6** Chemical constituents of the coal tar pitch extracts

| ID | Retention time  （min） | Rate（%） | Compound name | Category |
| --- | --- | --- | --- | --- |
| 1 | 15.105 | 1.991 | Phenanthrene | PAHs |
| 2 | 16.573 | 0.498 | Benzo[g]isoquinoline | heterocyclic compounds |
| 3 | 16.988 | 4.512 | 9H-Carbazole-9-methanol | heterocyclic compounds |
| 4 | 21.219 | 0.547 | 3-Methylcarbazole | heterocyclic compounds |
| 5 | 25.457 | 11.357 | Fluoranthene | PAHs |
| 6 | 26.065 | 0.814 | Indeno(1,2,3-ij)isoquinoline | heterocyclic compounds |
| 7 | 26.861 | 0.585 | Acenaphtho(1,2-b)pyridine | heterocyclic compounds |
| 8 | 27.503 | 10.106 | Pyrene | PAHs |
| 9 | 27.715 | 0.724 | Pyrene, 4, 5-dihydro^-^ | PAHs |
| 10 | 29.110 | 4.734 | 4-Azapyrene | heterocyclic compounds |
| 11 | 30.467 | 2.568 | 4H-Benzo[def]carbazole | heterocyclic compounds |
| 12 | 31.263 | 3.639 | 8-Methyl-4-azafluorenone | heterocyclic compounds |
| 13 | 31.591 | 0.523 | 7H-Benzo[c]carbazole | heterocyclic compounds |
| 14 | 32.074 | 0.427 | 7H-Benzanthrene | PAHs |
| 15 | 35.906 | 1.557 | 1-Methyl-acridone | heterocyclic compounds |
| 16 | 36.218 | 1.893 | 4-Methyl-acridone | heterocyclic compounds |
| 17 | 38.107 | 0.663 | Cyclopenta[cd]pyrene | PAHs |
| 18 | 38.662 | 1.498 | Benz[c]acridine | heterocyclic compounds |
| 19 | 39.015 | 0.764 | 7H-Benz[de]anthracen-7-one | PAHs |
| 20 | 40.362 | 2.039 | Benzo(a)acridine | heterocyclic compounds |
| 21 | 40.536 | 3.775 | Benz[a]anthracene | PAHs |
| 22 | 40.762 | 2.282 | Triphenylene | PAHs |
| 23 | 40.850 | 1.784 | Naphthacene | PAHs |
| 24 | 41.096 | 8.936 | 11H-Indeno(1,2-b)quinoline | heterocyclic compounds |
| 25 | 42.060 | 0.880 | Dibenzo[c,h][2,6]naphthyridine | heterocyclic compounds |
| 26 | 43.134 | 2.350 | Benzo(c)carbazole | heterocyclic compounds |
| 27 | 43.559 | 5.607 | 11H-Benzo[a]carbazole | heterocyclic compounds |
| 28 | 44.355 | 3.293 | 5(4H)-Thebenidinone | heterocyclic compounds |
| 29 | 45.559 | 0.743 | 2,6-Diphenylpyridine | heterocyclic compounds |
| 30 | 50.256 | 0.608 | Benz(a)anthracene-7-carbonitrile | PAHs |
| 31 | 51.714 | 4.283 | Benzo[a]pyrene | PAHs |
| 32 | 51.993 | 0.843 | Benzo[k]fluoranthene | PAHs |
| 33 | 54.168 | 3.140 | Benzo[e]pyrene | PAHs |
| 34 | 54.621 | 1.238 | Benzo[b]fluoranthene | PAHs |
